# High carrier frequency of pathogenic PATL2 gene mutations predicted in population: a bioinformatics-based approach

**DOI:** 10.3389/fgene.2023.1097951

**Published:** 2023-05-15

**Authors:** Hao Zhou, Ye-Lan Cai, Qing Luo, Lian Zou, Yong-Xiang Yin, Ying Chen, Fang Xiong

**Affiliations:** ^1^ Faculty of Science, The University of Sydney, Sydney, NSW, Australia; ^2^ Wuxi School of Medicine, Jiangnan University, Wuxi, China; ^3^ Reproduction Center, Wuxi Maternal and Child Health Hospital, Wuxi School of Medicine, Jiangnan University, Wuxi, China; ^4^ Pathology Department, Wuxi Maternal and Child Health Hospital, Wuxi School of Medicine, Jiangnan University, Wuxi, China; ^5^ Institute of Medical Genetics, Wuxi Maternal and Child Health Hospital, Wuxi School of Medicine, Jiangnan University, Wuxi, China

**Keywords:** infertility, *PATL2* mutation, bioinformatic analysis, single nucleotide polymorphism, assisted reproductive technology, germinal vesicle, empty follicle syndrome

## Abstract

Topoisomerase II homologue 2 (*PATL2*) has been confirmed to be a key gene that contributes to oocyte maturation. However, the allele distribution and carrier frequency of these mutations remain uncharacterized. So a bioinformatics subcategory analysis of *PATL2* mutations from outcome data and Single Nucleotide Polymorphism (SNP) databases was conducted. Altogether, the causative *PATL2* mutation number detected in patients with oocyte maturation defects in the clinical studies and pathogenic *PATL2* mutation sites predicted by software based on the database was approximately 53. The estimated carrier frequency of pathogenic mutation sites was at least 1.14‰ based on the gnomAD and ExAC database, which was approximately 1/877. The highest frequency of mutations detected in the independent patients was c.223-14_223-2del13. The carrier frequency of this mutation in the population was 0.25‰, which may be a potential threat to fertility. Estimated allele and carrier frequency are relatively higher than those predicted previously based on clinical ascertainment. A review of *PATL2* mutation lineage identified in 34 patients showed that 53.81%, 9.22% and 14.72% of the oocytes with *PATL2* mutations were arrested at the germinal vesicle (GV) stage, metaphase I (MI) stage and first polar body stage, respectively. Oocytes that could develop to the first polar body stage were extremely rare to fertilise, and their ultimate fate was early embryonic arrest. Phenotypic variability is related to the function of the regions and degree of loss of function of PATL2 protein. A 3D protein structure changes predicted by online tools, AlphaFold, showed aberrations at the mutation sites, which may explain partially the function loss. When the mutated and wild-type proteins are not in the same amino acid category, the protein structure will be considerably unstable. The integration of additional mutation sites with phenotypes is helpful in drawing a complete picture of the disease. Bioinformatics analysis of *PATL2* mutations will help reveal molecular epidemiological characteristics and provide an important reference for new mutation assessment, genetic counselling and drug research.

## 1 Introduction

Infertility issues are faced by 15% of couples worldwide, and almost half are estimated to have a genetic background. At present, >200 casual genes have been identified for female infertility, and genes related to oocyte maturation defect (OOMD) such as *ZP1* (OOMD1), *TUBB8* (OOMD2), *ZP3* (OOMD3), *PATL2* (OOMD4), *WEE2* (OOMD5), *ZP2* (OOMD6), *PANX1* (OOMD7), *BTG4* (OOMD8), *TRIP13* (OOMD9), *REC114* (OOMD10), *ASTL* (OOMD11), *FBXO43* (OOMD12) and *TBPL2* have been discovered by high-throughput sequencing (Chen et al., 2017; Sang et al., 2018; Wang et al., 2020; Zhang et al., 2020; Zheng et al., 2020; Cao et al., 2021; Sang et al., 2021; Yang et al., 2021; Capalbo et al., 2022; Loeuillet et al., 2022; Maddirevula et al., 2022). Topoisomerase II homologue 2 (*PATL2*) is a major pathogenic gene that is associated with germinal vesicle (GV) arrest and oocyte cleavage defects. Since the first case of homozygous mutation of *PATL2* gene in a patient who suffered from assisted reproductive technology (ART) failure because of oocytes being arrested at the GV stage (Chen et al., 2017) was reported, subsequent genetic testing was largely carried out and obtained important results in patients with phenotypes of recurrent failure in *in vitro* fertilisation (IVF) and intracytoplasmic sperm injection (ICSI), empty follicle syndrome (EFS), high proportion of GV-arrested oocytes, extremely low fertilisation rate, abnormal oocyte morphology and abnormally large first polar body. However, OOMD is a rare disease, and scarce variations have been found in suspected patients (Maddirevula et al., 2017; Christou-Kent et al., 2018; Huang et al., 2018; Wu et al., 2019; Liu et al., 2020; Wang et al., 2021; Huang et al., 2022; Huo et al., 2022; Lei et al., 2022; Sun et al., 2022); therefore, limited mutants are available for molecular epidemiological analysis. Furthermore, the phenotype caused by *PATL2* gene mutation was found to be insidious among the population and even in infertile women who underwent ART. Men carrying homozygous mutants of *PATL2* did not exhibit fertility issues (Huang et al., 2018). Therefore, conducting an epidemiological analysis based on the outcome data and analysing the mutant distribution and carrier frequency of *PATL2* using a population-based single nucleotide polymorphism (SNP) database was necessary.

Previous studies have shown that *PATL2* is required in oocyte maturation in model organisms. *PATL2*, as a translation repressor, anchors to the mRNA binding protein (mRNP) granule and is expressed in a spatio-temporal pattern. *PATL2* is expressed within a short time when metaphase I-arrested oocytes (referred to as GV oocytes) resume meiosis. Oocyte maturation is characterised by GV breakdown (GVBD) and first polar body extrusion. *PATL2* expression gradually decreases with development and is almost undetectable after the extrusion of the first polar body. Subsequently, the function of the translation repressor in the embryo may be compensated by other proteins. Oocytes carrying *PATL2* mutants have shown a spectrum of phenotypes, including GV arrest, fertilisation difficulty or early embryo arrest. Some mutants, such as p.Ile318Thr, have displayed considerably less disruptive protein function than some other mutants, such as p.Arg262* and p.Tyr186*, which have led to the complete loss of function [reviewed by Fei and Zhou (2022)]. Therefore, the aim of the study was to explore the database for prospective analysis to provide data for further mutation-phenotype correlation studies in the future.

When a different amino acid being incorporated into the polypeptide, it may have a wide range of effects on protein function. Mutations can lead to changes in the structure of an encoded protein. Changes of protein structure can be used for prediction of pathogenicity. Actually the elucidation of the three-dimensional structure of proteins is one of the biggest challenges in biology. AlphaFold is an ideal online prediction tool for protein structure changes, recently developed based on artificial intelligence. Over 1,700,000 resolved proteins were used for training. Three-dimensional shape of proteins can be accurately predicted with amino acid sequences, using deep learning algorithms combined with tension control algorithms.

## 2 Materials and methods

### 2.1 Pathogenic analysis of mutation sites

The GeneCards database (https://www.genecards.org/), NCBI Gene Database (https://www.ncbi.nlm.nih.gov/), Ensembl database (https://ensembl.org) query sequence and mutations were used for this study. In addition, Sorting Intolerant From Tolerant (SIFT, RRID:SCR_012813, http://sift.bii.a-star.edu.sg/), Combined Annotation Dependent Depletion (CADD, RRID:SCR_018393, https://cadd.gs.washington.edu/), Rare Exome Variant Ensemble Learner (REVEL, https://www.genome.ucsc.edu/cgi-bin/hgTrackUi?db=hg19&g=revel), and MutationAssessor (RRID:SCR_005762, http://mutationassessor.org/) were used to analyse the pathogenicity. The NCBI whole genome sequencing database (gnomAD) and the Exome Aggregation Consortium (ExAC) database were probed for allele frequency. Mutation Assess Amino acid sequences of human PATL2 protein was compared to different species in multiple sequence alignments using Clustal Omega (RRID:SCR_001591, https://www.ebi.ac.uk/Tools/msa/clustalo/). CADD≥ 10 is the cutoff value. Evolutionary conservation of the variants among the species was analysed using ConSurf server (http://consurftest.tau.ac.il). The outcome data of the *PATL2* pathogenic mutants reported in the literature (Liu et al., 2021; Huang et al., 2022; Huo et al., 2022; Sun et al., 2022) were re-analysed.

### 2.2 3D protein structure prediction

To predict the 3D protein structure, Protein Data Bank (PDB, http://www.rcsb.org/), SWISS-MODEL (http://www.swissmodel.expasy.org), Polymorphism Phenotyping v2 tool (Polyphen - 2, http://genetics.bwh.harvard.edu/pph2) (Adzhubei et al., 2010) and AlphaFold Protein Structure Database (https://alphafold.com/) (Jumper et al., 2021; Varadi et al., 2022) were used and the results were compared.

## 3 Results

### 3.1 Molecular epidemiology and bioinformatics analysis of *PATL2* variants

#### 3.1.1 Phenotype spectrum in IVF/ICSI cases involving *PATL2* variants

The data on pathogenic *PATL2* mutants reviewed by Sun et al. and reported in a recent study were reanalysed (Liu et al., 2021; Huang et al., 2022; Huo et al., 2022; Sun et al., 2022). A total of 34 patients with OOMD were included. The reported average age of women was 31.41 ± 4.34, with an average infertility duration of 7.00 ± 3.04 years. A total of 84 IVF/ICSI cycles were recorded in 34 patients with 2.44 ± 1.61 as the average number of cycles. A total of 944 oocytes were retrieved, with an average of 11.24 ± 7.13 oocytes for each ART cycle. Among the 944 oocytes, 53.81% (508/944) were at the GV stage, 9.22% (87/944) were at metaphase I (MI), 14.72% (139/944) showed the first polar body (matured), and 22.25% (210/944) were empty follicles, exhibited atresia. Fertilisation and cleavage were extremely rare. None of the reported cases carrying the mutation located between p.342 and 446 (around exons 12 and 13) showed the first polar body. The cases in which oocytes developed into the first polar body were frequently found to carry a mutation at the N-terminus before position p.217. However, these oocytes failed to fertilise or were arrested at an early stage. Thus, complete loss-of-function mutations may prevent oocytes from developing into the first polar body phase. We conclude that phenotypic variability is related to the region of function and degree of loss of function of the PATL2 protein. Significant PATL2 protein impairment results in oocytes being arrested at an earlier stage.

#### 3.1.2 Mutation distribution of *PATL2* in the cases with OOMD

**FIGURE 1:**
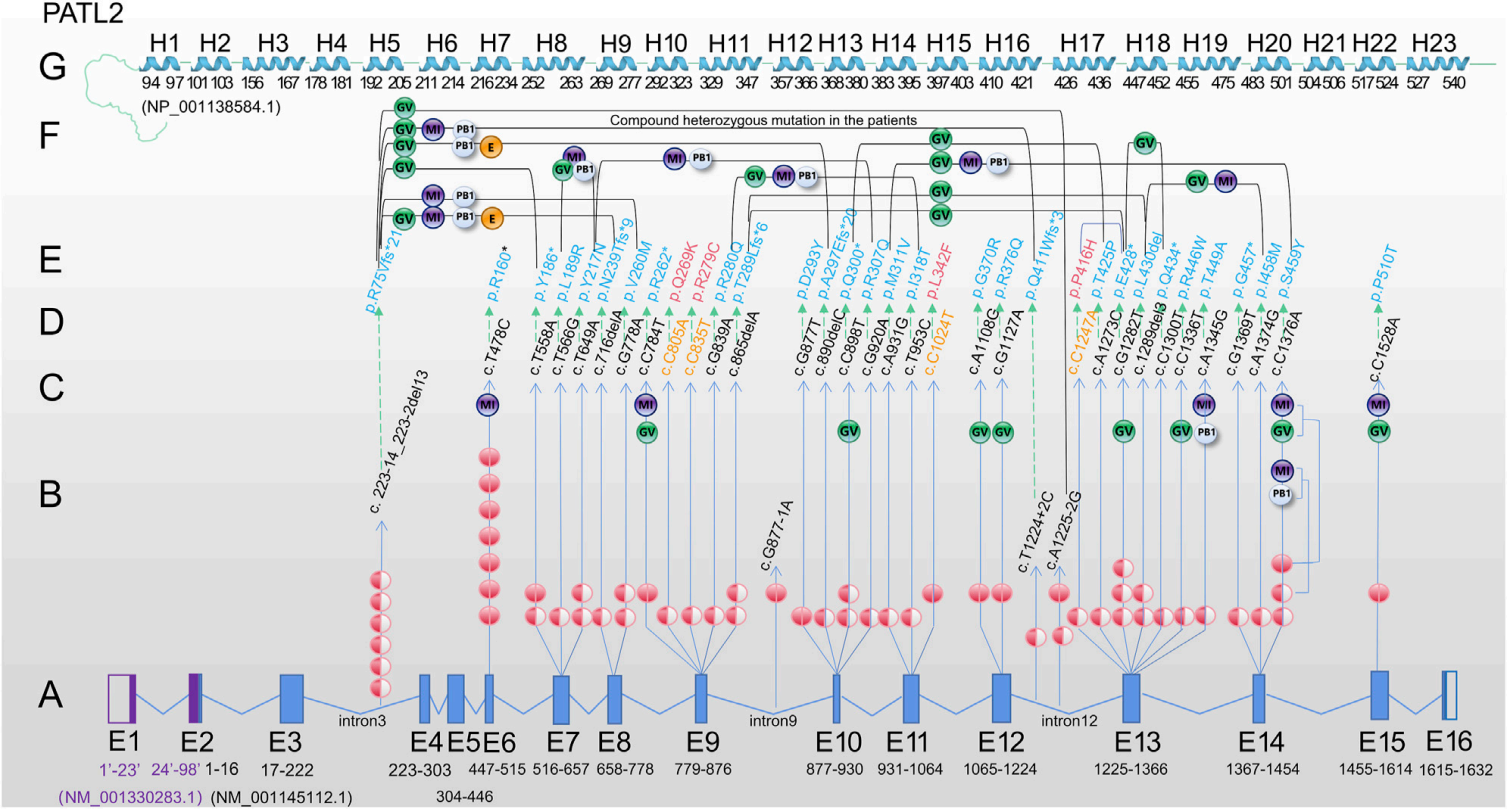
Review of *PATL2* mutation lineage identified in 34 patients characterized by oocyte maturation defect from the published paper. **(A)** PATL2 gene is a special gene that has a little difficulty in localising mutation in the exons. To facilitate localization, we mapped all 16 exons. E1 and E2 in purple are from NM_ 001,330,283.1; E3-E16 are from NM_ 001145112.1. The arrow points to the cDNA changes (column D) and protein changes (column E). **(B)** The number of independent patients with mutations is shown on the line with the arrow. The solid red ball represents homozygous mutation, and the semi-solid ball represents heterozygous mutation. **(C)** The developmental stage of oocytes in the patients carrying homozygous mutation or only one allele of the heterozygous mutation is shown in the upper end of the line. **(D)** cDNA changes. **(E)** Protein changes, including 31 mutation sites (in blue) reported in previous studies. **(F)** The developmental stage of oocyte/embryo in patients with biallelic compound heterozygous mutations. Two mutations are connected by a bridge line on which the oocyte/embryo are marked. **(G)** Linearly arranged H1-H23 alpha-helix region, which was manually designed according to the schematic diagram from the tertiary structure of PATL2 protein (NP_001,138,584.1) predicted by AlphaFold. The starting and ending positions are marked. Different software may have difference in prediction, which is only for reference. Note: PATL2 (PAT1 homologue 2), GV (oocyte arrested at the germinal vesicle stage), MI (oocyte arrested at metaphase I of meiosis), PB1 (first polar body oocyte), E (cleavage embryo).

We mapped 31 reported mutations onto the atomic model (Figure 1) and analysed the potential effects of these mutations, including 18 missense, 7 nonsense, 3 splicing and 3 deletion mutations. Among them, 74.19% (23/31), 67.74% (21/31) and 32.26% (10/31) were located in the mRNA decay factor PAT1 domain (p.252-491), helical region of PATL2 protein and non-helical regions or introns, respectively.

Based on the published articles (Chen et al., 2017; Huang et al., 2018; Wu et al., 2019; Liu et al., 2020; Huang et al., 2022), the c.223-14_223-2del13 mutation was the most frequently reported in different populations by different researchers, including 7 independent patients (Figure 1B). The other most frequent mutation was the homozygous mutation, c.T478C. Seven cases were found in two studies and one researcher contributed to six cases (Maddirevula et al., 2017). In addition, homozygous missense mutations (c.T478C, c.T558A, c.C784T, c.G877T, c.A1108G, c.G1127A, c.C1376A and c.C1528A) and homozygous splicing mutations (c.A1225-2G and c.G877-1A) were found in the patients. A total of 7 nonsense mutations (p.P160*, p.Y186*, p.R262*, p.Q300*, p.E428*, p.Q434* and p.G457*) were detected. Moreover the mutations at the exon region found in two or more cases in different populations were c.T478C, c.T558A, c.T649A, c.G778A, c.C898T, c.G1282T and c.C1376A and 4 splicing sites (c.223-14_223-2del13, c.T1224+2C, c.A1225-2G and c.G877-1A), indicating a certain distribution frequency in the population. We found that a relatively high frequency of compound heterozygosity was found in the c. 223-14_223-2del13 site. Other mutations associated with compound heterozygous mutations include c.T558A, c.T566G, c.T649A, c.716delA, c.G778A, c.G839A, c.865delA, c.G877T, c.C898T, c.G920A, c.A931G, c.T953C, c.T1224+2C, c.A1225+2C, c.C1247A, c.A1273C, c.G1282T, c.C1300T and c.C1376A. Only one heterozygous mutation was found, but the second mutation was not found within the exon and flanking sequences (c.T649A and c.G1282T), suggesting that there may be variations in the regulatory or intronic regions.

Based on the above exon distribution analysis, the exon with the highest number of mutation sites was revealed, including Exon13 (6 sites, 9 patients), Exon10 (4 sites, 5 patients), Exon14 (3 sites, 5 patients) and Exon7 (3 sites, 5 patients), Exon9 (3 sites, 4 patients). Exon13 is a 142 base pair exon that encodes 48 amino acids (p.409-456) and spans five helices. Seven out of 48 (14.58%) amino acids were altered to cause the phenotype. In small laboratories, hotspot sequencing has been suggested to reduce the burden on patients and technicians, resulting in economic benefits.

#### 3.1.3 SNP allele frequency analysis on population

##### 3.1.3.1 SNP allele frequency over the reported cases

We searched for the frequency of SNP alleles and 20 loci with SNP accession numbers in the reported cases. We found that the frequency of a single site was considerably low, some of which were only 1/150,000. The top three were c.G839A (0.00519), c.C805A (0.00340) and C.T478C (0.00306). The cumulative allele frequency of these loci was 48.09 per 100,000 people, which was approximately 1/2080.

##### 3.1.3.2 Pathogenicity prediction and allele frequency analysis in the population database

**FIGURE 2:**
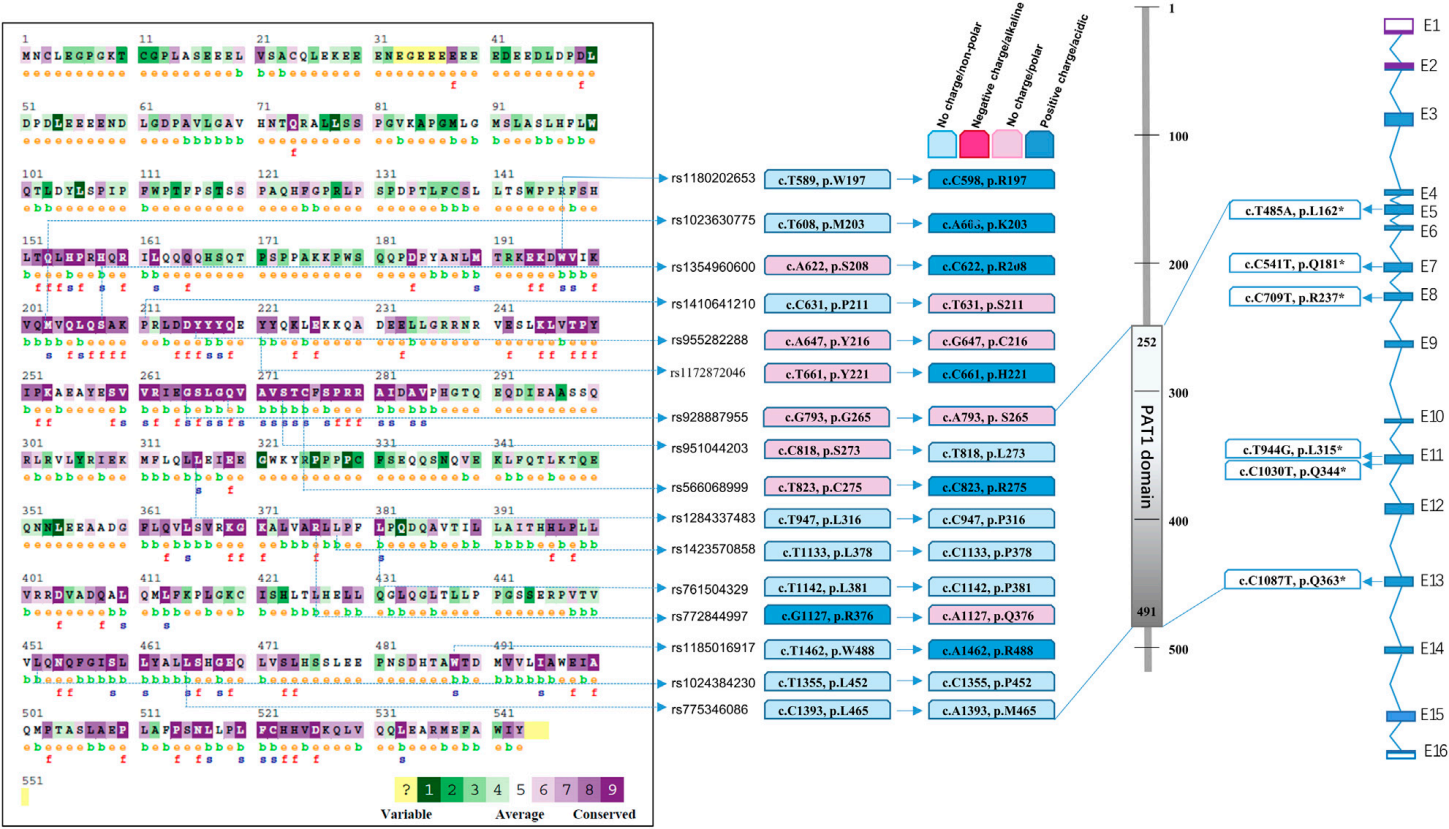
Spectrum of predicted pathogenic mutations of *PATL2* based on public SNP database. Comparison of conservation among species was conducted using the ConSurf online database. Different colour blocks represent the conservation levels. A total of 16 predicted missense mutations and 6 stop codon changes in cDNA and protein are shown and located.

A public database was obtained by whole-genome sequencing and whole-exome sequencing from all over the world. In addition to the mutation sites from the patients summarised in Figure 1, a total of 16 SNPs (Figures 2A, B) were predicted and labelled to be pathogenic by at least four software programs, among which 10 SNPs were located in the PAT1 domain p.252-491 (Figure 2C). The cumulative carrier frequency of these SNPs was approximately 64.86 per 100,000 individuals. As the number of cases increased, these loci were likely to be detected.

We performed statistical analysis of nonsense mutations and mutations predicted to be pathogenic using more than four software programs. In addition to the phenotypic seven nonsense mutations summarised in Figure 1, we searched for six mutations with stop codons (Figure 2D) which were recorded in Uniprot, including rs760151963 (c.T485A, p.L162*), rs1281934664 (c.C541T, p.Q181*), rs763592153 (c.C709T, p.R237*), rs1457057185 (c.T944G, p.L315*), rs1176737926 (c.C1030T, p.Q344*) and rs751701388 (c.C1087T, p.Q363*), and the incidence of these sites was extremely low. The cumulative carrier frequency of these nonsense mutations was approximately 3.81 per 100,000 individuals.

Thus, the cumulative allele frequency of the reported mutation sites, together with 22 mutations based on the public database, was estimated to be 1.14‰, which indicated that one in 877 people might be a carrier.

#### 3.1.4 Conservation analysis among species

Multiple sequence alignments of the amino acid sequence of the PATL2 protein were compared among species using Clustal Omega (https://www.ebi.ac.uk/Tools/msa/clustalo/). The comparison map was marked using ConSurf (Figure 2A). Comparative analysis revealed conservation in most of the predicted SNPs screened from the public databases (Figures 2A, B). Among the 22 pathogenic SNPs predicted by the software, loci rs1023630775 (c.T608A) and rs1354960600 (c.A622C) were located in the conserved region (p.201-285, p.451-530).

Mutation from the wild type into amino acids of different groups that have different properties may disrupt protein stability, resulting in incorrect folding and unstable 3D architecture. Both the alpha-helix and beta-strand structures have their own tendency towards amino acids, and some of them have unique patterns. For example, mutation of the positive charge into no charge (c.T608A, p.M203K) directly destroys the interaction with other amino acids and subsequently may lead to issues in folding. If the hydrophobic property is converted into polar property, the structure is affected. Furthermore, some amino acids are inherently dangerous, such as proline (c.T1133C, p.L378P), because proline is an imino acid with a stable angle, and mutations in proline will change the protein structure to a large extent. In addition, mutation in glycine (c.G1108A, p.G370R) leads to the R group (hydrogen atom) gaining extensive freedom, thereby affecting the stability of the protein structure.

### 3.2 Analysis of the structural changes in mutant proteins

#### 3.2.1 The map of mutation distribution in domains

According to the distribution map of mutation sites summarised here, all mutations were located in the FUGUE (CATH-based) domain (p.158-534). The percentages of mutations located in the mRNA decay factor PAT1 domain (p.252-491), at the N-terminal region (p.1-239) and at the C-terminal region (p.492-543) were 70.97% (22/31), 16.13% (5/31) and 3.23% (1/31), respectively. Of these, 67.74% (21/31) were located in the alpha helix predicted by the AlphaFold Protein Structure Database. The percentage of stop codon mutations with truncated proteins which were all located in the FUGUE (CATH-based) domain was 22.58% (7/31).

#### 3.2.2 Prediction of secondary and tertiary structures of mutant proteins

**FIGURE 3:**
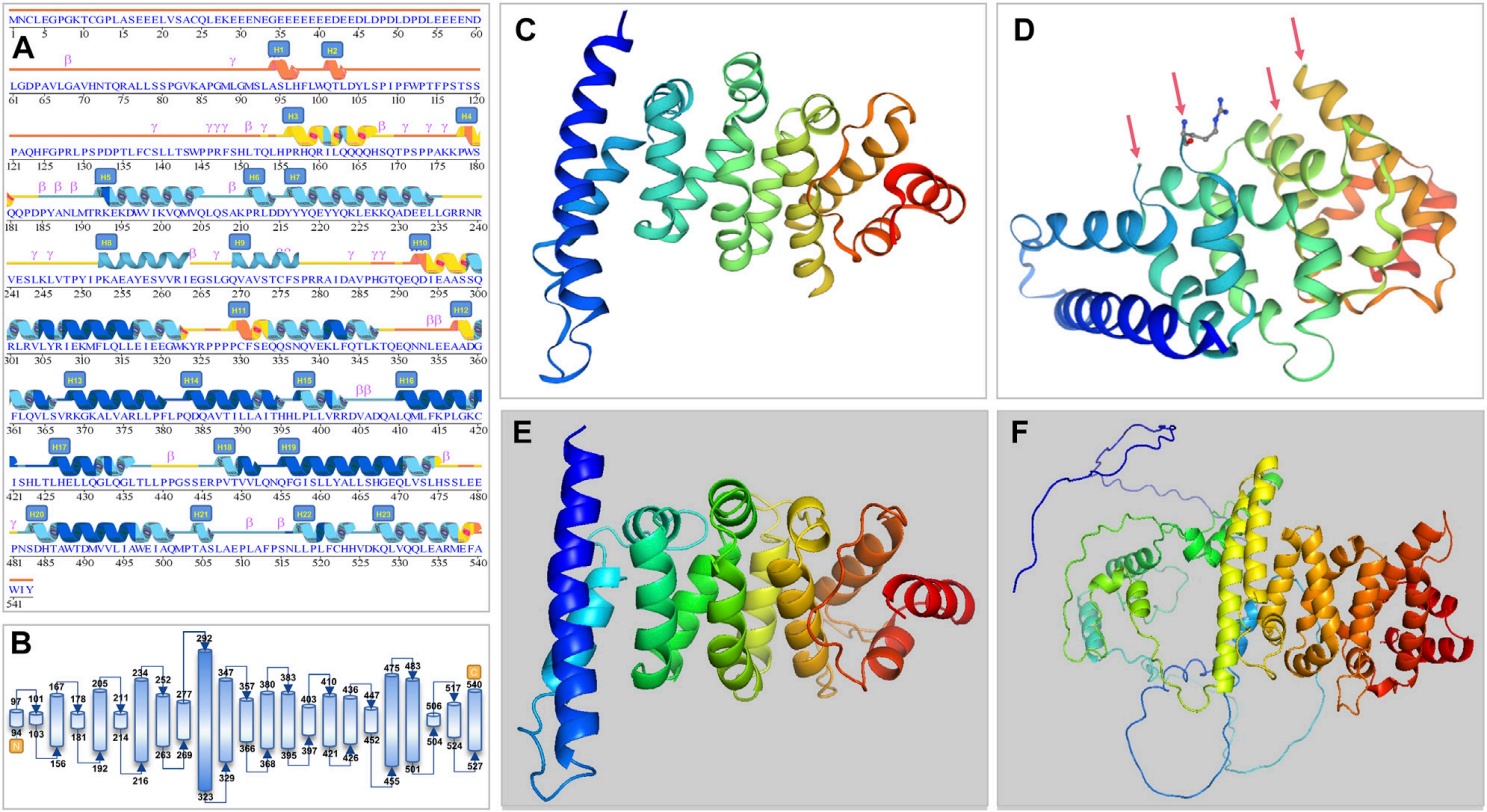
Secondary and tertiary crystal structure prediction diagram of the PATL2 protein. **(A)** Prediction by AlphaFold. We have revised some alpha-helix areas and serial numbers and have added H9 (p.252-263) and H10 (p.269-277) helices according to the tertiary structure. **(B)** Schematic diagram of protein structure showing the start and end position of the helix. **(C,D)** are 3D structure predicted by SWISS-MODEL. Arrows point to the four breakpoints because of unmatched sequence. **(E)** 3D structure predicted by Pyren-2 with only a part of the crystal shown (p.298-536; 239aa). **(F)** The whole length of PATL2 of 543 amino acids as predicted by AlphaFold.

The PATL2 protein sequence (NP_001138584.1) was first added in the SWISS-MODEL and Polymorphism Phenotyping 2.0 (Pyren-2) tool to obtain the predicted crystal of the PATL2 protein fragment (Figures 3C–E). The PANX1 protein fragment (p.299-540; 247aa) predicted by SWISS-MODEL is homologous to the PAT1 homologue 1 (SMTL ID: 2xer.1.A). Compared to its sequence, PATL2 has 10 inconsistent amino acids with four free breakpoints in the tertiary structure (Figure 3D). The PATL2 fragment predicted by Pyren-2 had no breakpoint, but only a part of the crystal was found (p.298-536; 239aa).

**FIGURE 4:**
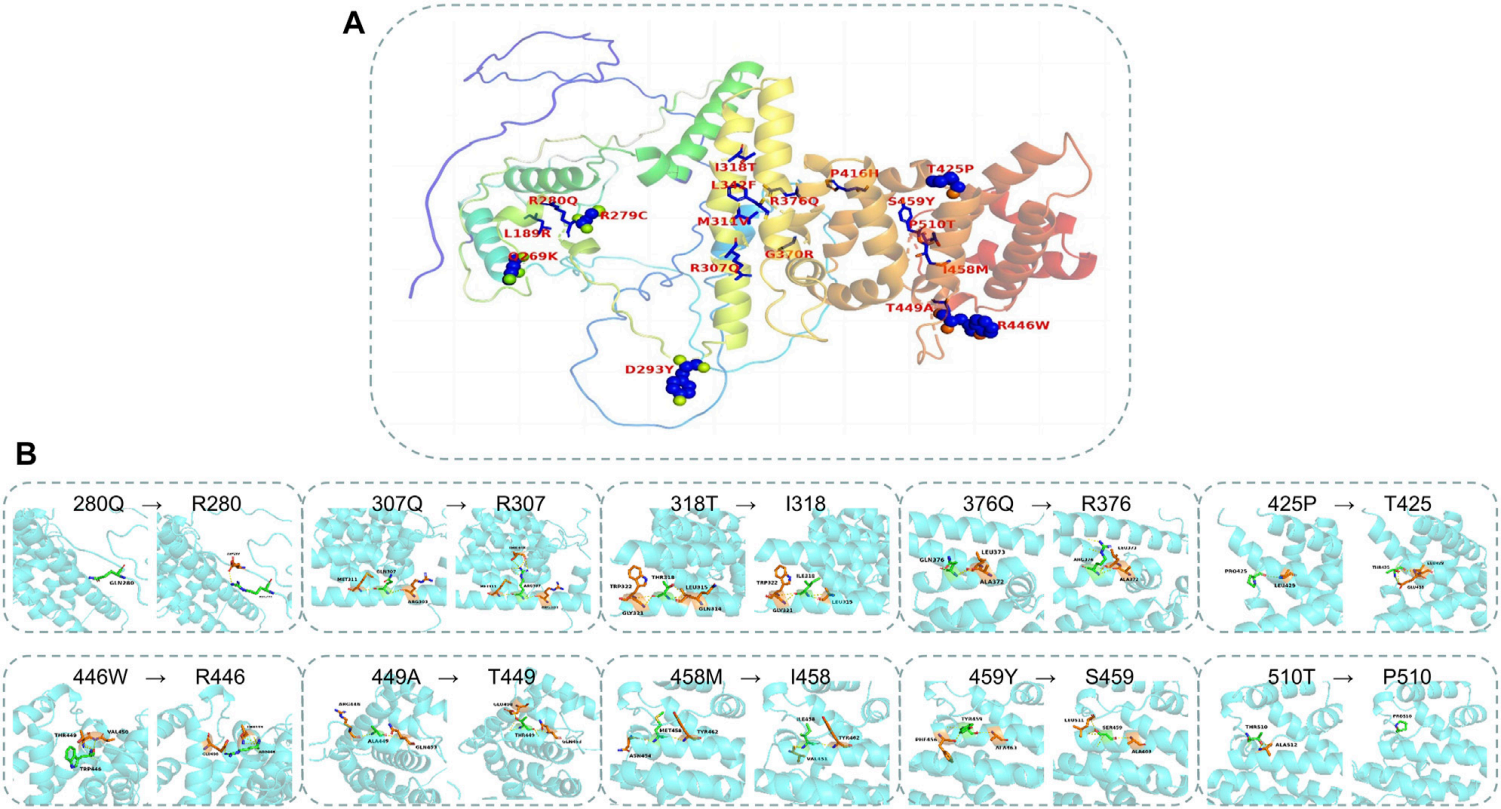
Mapping the mutations in the PATL2 crystal. **(A)** The distribution of 18 missense mutations detected from the patients. **(B)** The structure and binding changes between mutant and wild type.

The AlphaFold Protein Structure Database was used to make predictions with minor modifications (Figures 3A, B, F). The free AlphaFold database is an artificial intelligence system developed by DeepMind that predicts a protein’s 3D structure from an amino acid sequence. DeepMind is used to train the neural network to predict the distance between amino acid pairs and the angle of the chemical bond. It regularly achieves an accuracy competitive with the experiment. A full-length protein structure was produced, which was more accurate than that produced by the previous prediction technology. A total of 18 mutation sites were aligned in the 3D structure of PATL2 protein (Figure 4A). The distribution of the mutant sites is easily seen scattered in the different regions of the protein. Most of them are located in the helix regions. Of them ten mutants having obvious structure or binding changes compared to the wild type are demonstrated in Figure 4B. For example, the amino acid 458 of the protein mutates from ILE to MET, which leads to the change of the force among residues. There is interaction between ILE and the other three residues. After mutated to MET, only two residues have interaction with it, and the residues also change. There is hydrogen bond force between ILE458 and VAL451, and there is hydrogen bond force between MET and ASN454. Some mutations result in low hydrogen bond force between amino acid residues, such as R280Q. A hydrogen bond is formed between ARG280 and ASP283 before mutation, but after mutation to GLN there is no charge.

## 4 Discussion

Populations in different regions have experienced a long history of integration and show a certain genetic distribution. With the development of high-throughput sequencing, an increasing number of human samples have been sequenced, and various infertility-related genes and loss-of-function mutations have been identified and confirmed (Biswas et al., 2021; Houston et al., 2021; Jiao et al., 2021). Particularly, when infertile patients with recurrent IVF/ICSI failure or with a family history sought help from genetic testing, pathogenic genes and loci quickly accumulated, together with a large number of loci of possibly pathogenic or unknown clinical significance, waiting to reveal their potential functions and effects. A prospective assessment based on public databases and analyses is necessary.

The carrying rate of suspected pathogenic alleles of *PATL2* in the global population has not been estimated. Based on a public database of more than 300,000 whole genomes and whole exome sequencing, we predicted for the first time that the overall carrier frequency of suspected pathogenic mutation sites is at least 1.14‰, which is 1/877. This study summarised 53 mutations in *PATL2*, including 31 mutations from reported independent individuals, six nonsense mutations and 16 predicted pathogenic mutations identified from the public database, which expanded the spectrum of genetic causes of oocyte maturation defects. Although 22 loci from the public database was not reported in the infertile population, they might be found in ART patients undergoing genetic testing as the sample size increases. Subsequent studies are recommended to confirm whether these mutations have true influence on oocyte maturation. It is believed that this carrier frequency will continue to be rewritten when additional sequencing results are shared, additional clinical cases are tested and confirmatory experiments are performed. Identifying more casual mutants will help establish the complete mapping of genetic factors in human infertility.

Analysing the distribution of these mutations showed that the highest frequency of mutations detected in the independent patients was c.223-14_223-2del13. The carrier frequency in the population was 0.25‰, which may be a potential threat to fertility. Exons with additional mutation sites and cases were Exon13 (6 sites, 9 patients). This study carried out a molecular epidemiological analysis based on the updated definition of the PAT1 domain of the PATL2 protein within p.252-491. Analysis of 31 *PATL2* compound heterozygous and homozygous mutation sites in patients with oocyte maturation defects showed that 70.91% of mutations were located in the PAT1 domain, indicating the important function of the RNA-binding domain. Identifying the regulatory RNA that can bind to this domain is an important future research goal.

We predicted the entire secondary and tertiary structures of the PATL2 protein. To the best of our knowledge, we mapped for the first time 23 helical regions on the secondary and tertiary structures of the PATL2 protein. A percentage of 65.71% of the mutation sites detected in the reported patients were in the helical region, and the rest were in the non-helical region. A percentage of 81.82% of the SNP identified in the public database were located in the helical region and 18.18% were located in the non-helical region, indicating the importance of changes in amino acid in helical regions. According to the polarity of the side chain, amino acids can be divided into four categories: polar with a positive charge, polar with a negative charge, polar without a charge and non-polar. Each amino acid has a specific R side chain, which determines its physical and chemical properties. The solubility of non-polar to polar amino acids in water increases. The mutants causing non-polar to polar changes with negative charges include two sites (p.L189R and p.P416H) and the ones causing changes from non-polar hydrophobic to polar uncharged include two sites (p.I318T and p.P510T). The amino acids in the polar uncharged (hydrophilic) side chain contain neutral polar residues; therefore, when the polar uncharged amino acids are between hydrophobic amino acids and charged amino acids, they can form hydrogen bonds with water molecules to change the protein structure. Contrastingly, the hydrogen bond breaks when polar and uncharged amino acids become non-polar amino acids (such as p.T425P and p.T449A). Additionally, proline is an imino acid with a stable angle; therefore, the mutation of 425T to proline will greatly change the structure of the protein itself. Sites that change from polar uncharged to polar negative charge (e.g., p.G370R), from polar negative charge to polar uncharged (e.g., p.R279C, p.R280Q, p.R307Q and p.R376Q) and from polar positive charged to polar uncharged (p.D293Y) were found. Generally speaking, if wild-type and mutated amino acid do not belong to the same category, the protein structure is unstable.

The analysis of 944 oocytes reported in independent individuals diagnosed with oocyte maturation defects in ART cycles showed a phenotype spectrum: 53.81% of the oocytes were arrested at the GV stage, 9.22% at the MI stage, 15.36% at the first polar body stage and 21.61% exhibited atresia and had empty follicles. Even those carrying the same compound heterozygous or homozygous mutations at the same locus showed different phenotypes (Wu et al., 2019). The expression of *PATL2* in granulosa cells of polycystic ovary patients was reported to have significantly increased (Peng et al., 2021). Although studies on the factors that influence PATL2 are rare, *PATL2* mutants may be sensitive to other factors, such as the follicular microenvironment and external environment, at the early stage of meiotic recovery, resulting in differences in phenotypic profiles. ICSI oocytes failed to establish pregnancy, even though they were fertilised and cleaved. Thus, even if development reaches the first polar body stage, ICSI may not alter embryo fate. Although *PATL2* expression is almost undetectable after extrusion of the first polar body in normal oocytes, early dysfunction has a subsequent effect on oocyte outcome. Recently, the results of the study by Hatirnaz and team provided the hope of pregnancy for patients with oocyte maturation abnormalities (Hatirnaz et al., 2022). According to the Hatirnaz and Dahan Classification System, the ongoing pregnancy in women with type II (MI arrest) and type V (GV, MI and MII mixed arrest) oocyte seemed to be achieved by dual stimulation in *in vitro* maturation (Duastim IVM) in the Hartinaz clinic; this did not include patients with Type I (GV arrest), Type III (MII arrest) and Type IV (GV-MI arrest) oocyte abnormalities. One patient had two failed IVF attempts because of GV arrest by *PANX1* mutation and was treated with letrozole priming IVM, and one fertilised embryo was obtained, giving the *PANX1* mutation oocyte a living potential. Therefore, this led to an important step forward in therapy (Hatirnaz et al., 2022). But at present the patients with pathogenic mutations are still suggested to stop the recurrent self oocyte attempts and refer to a oocyte donation strategy. For those with only one heterozygous mutation, it should also be noted, since biallelic gene effect may also reduce the fertility capacity largely.

Mouse is a good model for functional analysis when a novel mutation is found. But the phenotypes are not always same. It was found that Patl2^−/−^ female mice exhibited influence on fertility not as severe as that in human, the Patl2 −/− oocytes could fertilise and develop to blastocyst stage. But the absence of Patl2 affected oocyte growth which showed abnormal small size of the GV oocytes and MII oocytes. Abnormal spindle morphology, misalignment of chromosomes on the spindle and numerous cytoplasmic asters were also notably found in the arrested oocytes (Christou-Kent et al., 2018). There may exist the other repair mechanism to overcome the barrier in oocyte development in mice.

Recently, it was reported that an induced pluripotent stem cell (iPSC) model was constructed from CD34^+^ cells in the peripheral blood of a 28-year-old patient with *PATL2* compound heterozygous mutation, and was reprogrammed (Xue et al., 2022). iPSCs provide the possibility for in-depth research on their mechanisms and subsequent targeted therapies. Additionally, the PATL2^Y217N^ mutant was transfected into 293T cells to explore the possible mechanism by which *PATL2* mutations cause oocyte maturation defects. The mutant seemed to be more stable than PATL2^WT^, resulting in abnormal accumulation of the PATL2 protein. It is worth noting that mouse oocytes microinjected with PATL2^Y217N^ cRNA showed decreased ubiquitination levels together with abnormally large polar bodies similar to those in patients. This phenomenon is similar to the phenotype observed in mouse MOS −/− oocytes, with anomalous polar bodies, partial symmetrical cleavage and abnormal spindles (Cao et al., 2021). Spatiotemporal and specific expression of PATL2 at the meiosis initiation stage and proper functioning of the downstream gene inhibition at an appropriate time (after the first polar body is extruded) are particularly important. Too little or too much untimely expression or accumulation has a severe influence on oocyte maturation. The interference of the above mutations in the process of early maturation allows us to consider the method to choose the appropriate time to correct the defect when designing a targeted therapy. Therefore, the choice of time, mode and dose may become a bottleneck for successful treatment and intervention.

In summary, bioinformatics analysis of *PATL2* gene mutations will help reveal the molecular epidemiological characteristics, help identify the prevalence of *PATL2* gene mutations and provide important references for new mutation assessment, function prediction, phenotype prediction, disease diagnosis, genetic counselling, drug research and incubation and formulation of intervention strategies. Furthermore, it provides a reference for a reasonable sample size involved in epidemiological studies of *PATL2* gene mutations. Using the existing database for prediction analysis can reduce the number of discovery and confirmation cycles for novel loci. Patient-derived mutated animal models are recommended to be used for establishing whether these features also reappear in the model animal and for exploring the mechanisms underlying the phenotypes involved in structural defects and infertility.

## Data Availability

The original contributions presented in the study are included in the article/supplementary material, further inquiries can be directed to the corresponding authors.

## Abbreviations

ASTL, astacin like metalloendopeptidase; BTG4, BTG anti-proliferation factor 4; EFS, empty follicle syndrome; FBXO43, F-box protein 43; GV, germinal vesicle; MI: GVBD, Germinal vesicle breakdown; ICSI, intracytoplasmic sperm injection; IVF, *in vitro* fertilization; MI, Metaphase I; mRNP, mRNA binding protein; OOMD, oocyte maturation defect; PANX1, pannexin 1; PATL2, Topoisomerase II homologue 2; PB1, first polar body; REC114, REC114 meiotic recombination protein; SNP, single nucleotide polymorphism; TBPL2, TATA-box binding protein like 2; TRIP13, thyroid hormone receptor interactor; TUBB8, tubulin beta 8 class VIII;(OOMD2); WEE2, WEE2 oocyte meiosis inhibiting kinase; ZP1, zona pellucida-1; ZP2, zona pellucida-2; ZP3, zona pellucida**-**3.

## References

[B1] Adzhubei I. A. Schmidt S. Peshkin L. Ramensky V. E. Gerasimova A. Bork P. (2010). A method and server for predicting damaging missense mutations. Nat. Methods. 7 (4), 248–249. 10.1038/nmeth0410-248 20354512 PMC2855889

[B2] Biswas L. Tyc K. El Yakoubi W. Morgan K. Xing J. Schindler K. (2021). Meiosis interrupted: The genetics of female infertility via meiotic failure. Reproduction 161 (2), R13–R35. 10.1530/REP-20-0422 33170803 PMC7855740

[B3] Cao Q. Zhao C. Wang C. Cai L. Xia M. Zhang X. (2021). The recurrent mutation in PATL2 inhibits its degradation thus causing female infertility characterized by oocyte maturation defect through regulation of the mos-MAPK pathway. Front. Cell Dev. Biol. 9, 628649. 10.3389/fcell.2021.628649 33614659 PMC7890943

[B4] Capalbo A. Buonaiuto S. Figliuzzi M. Damaggio G. Girardi L. Caroselli S. (2022). Maternal exome analysis for the diagnosis of oocyte maturation defects and early embryonic developmental arrest. Reprod. Biomed. Online. 45 (3), 508–518. 10.1016/j.rbmo.2022.05.009 35798635

[B5] Chen B. Zhang Z. Sun X. Kuang Y. Mao X. Wang X. (2017). Biallelic mutations in PATL2 cause female infertility characterized by oocyte maturation arrest. Am. J. Hum. Genet. 101 (4), 609–615. 10.1016/j.ajhg.2017.08.018 28965849 PMC5630194

[B6] Christou-Kent M. Kherraf Z. E. Amiri-Yekta A. Le Blevec E. Karaouzene T. Conne B. (2018). PATL2 is a key actor of oocyte maturation whose invalidation causes infertility in women and mice. EMBO Mol. Med. 10 (5), e8515. 10.15252/emmm.201708515 29661911 PMC5938616

[B7] Fei C. F. Zhou L. Q. (2022). Gene mutations impede oocyte maturation, fertilization, and early embryonic development. Bioessays 44 (10), e2200007. 10.1002/bies.202200007 35900055

[B8] Hatirnaz S. Hatirnaz E. Çelik S. Çalışkan C. S. Tinelli A. Malvasi A. (2022). Unraveling the puzzle: Oocyte maturation abnormalities (OMAS). Diagn. (Basel) 12 (10), 2501. 10.3390/diagnostics12102501 PMC9601227 36292190

[B9] Houston B. J. Riera-Escamilla A. Wyrwoll M. J. Salas-Huetos A. Xavier M. J. Nagirnaja L. (2021). A systematic review of the validated monogenic causes of human male infertility: 2020 update and a discussion of emerging gene-disease relationships. Hum. Reprod. Update. 28 (1), 15–29. 10.1093/humupd/dmab030 34498060 PMC8730311

[B10] Huang L. Tong X. Wang F. Luo L. Jin R. Fu Y. (2018). Novel mutations in PATL2 cause female infertility with oocyte germinal vesicle arrest. Hum. Reprod. 33 (6), 1183–1190. 10.1093/humrep/dey100 29697801

[B11] Huang L. Wang Y. Lu F. Jin Q. Song G. Ji J. (2022). Novel mutations in NLRP5 and PATL2 cause female infertility characterized by primarily oocyte maturation abnormality and consequent early embryonic arrest. J. Assist. Reprod. Genet. 39 (3), 711–718. 10.1007/s10815-022-02412-4 35091966 PMC8995404

[B12] Huo M. Zhang Y. Shi S. Shi H. Liu Y. Zhang L. (2022). Gene spectrum and clinical traits of nine patients with oocyte maturation arrest. Front. Genet. 13, 772143. 10.3389/fgene.2022.772143 35140748 PMC8819080

[B13] Jiao S. Y. Yang Y. H. Chen S. R. (2021). Molecular genetics of infertility: Loss-of-function mutations in humans and corresponding knockout/mutated mice. Hum. Reprod. Update. 27 (1), 154–189. 10.1093/humupd/dmaa034 33118031

[B14] Jumper J. Evans R. Pritzel A. Green T. Figurnov M. Ronneberger O. (2021). Highly accurate protein structure prediction with AlphaFold. Nature 596 (7873), 583–589. 10.1038/s41586-021-03819-2 34265844 PMC8371605

[B15] Lei Q. Li J. Zhou X. Zhang W. (2022). Oocyte maturation arrest due to compound heterozygous variants of the PATL2 gene in a case. Zhonghua. Yi. Xue. Yi. Chuan. Xue. Za. Zhi. 39 (7), 759–762. 10.3760/cma.j.cn511374-20210316-00234 35810437

[B16] Liu M. Du H. Song D. Si F. Ma L. Wang Y. (2021). Relationship of symptom stress, care needs, social support, and meaning in life to quality of life in patients with heart failure from the acute to chronic stages: A longitudinal study. Zhonghua Sheng Zhi Yu Bi Yun Za Zhi 41 (3), 252–256. 10.1186/s12955-021-01885-8 PMC8572479 34742311

[B17] Liu Z. Zhu L. Wang J. Luo G. Xi Q. Zhou X. (2020). Novel homozygous mutations in PATL2 lead to female infertility with oocyte maturation arrest. J. Assist. Reprod. Genet. 37 (4), 841–847. 10.1007/s10815-020-01698-6 32048119 PMC7183019

[B18] Loeuillet C. Dhellemmes M. Cazin C. Kherraf Z. E. Fourati Ben Mustapha S. Zouari R. (2022). A recurrent ZP1 variant is responsible for oocyte maturation defect with degenerated oocytes in infertile females. Clin. Genet. 102 (1), 22–29. 10.1111/cge.14144 35460069 PMC9327729

[B19] Maddirevula S. Coskun S. Al-Qahtani M. Aboyousef O. Alhassan S. Aldeery M. (2022). ASTL is mutated in female infertility. Hum. Genet. 141 (1), 49–54. 10.1007/s00439-021-02388-8 34704130

[B20] Maddirevula S. Coskun S. Alhassan S. Elnour A. Alsaif H. S. Ibrahim N. (2017). Female infertility caused by mutations in the oocyte-specific translational repressor PATL2. Am. J. Hum. Genet. 101 (4), 603–608. 10.1016/j.ajhg.2017.08.009 28965844 PMC5630161

[B21] Peng S. L. Wu Q. F. Xie Q. Tan J. Shu K. Y. (2021). PATL2 regulated the apoptosis of ovarian granulosa cells in patients with PCOS. Gynecol. Endocrinol. 37 (7), 629–634. 10.1080/09513590.2021.1928066 34008465

[B22] Sang Q. Li B. Kuang Y. Wang X. Zhang Z. Chen B. (2018). Homozygous mutations in WEE2 cause fertilization failure and female infertility. Am. J. Hum. Genet. 102 (4), 649–657. 10.1016/j.ajhg.2018.02.015 29606300 PMC5985286

[B23] Sang Q. Zhou Z. Mu J. Wang L. (2021). Genetic factors as potential molecular markers of human oocyte and embryo quality. J. Assist. Reprod. Genet. 38 (5), 993–1002. 10.1007/s10815-021-02196-z 33895934 PMC8190202

[B24] Sun L. Tong K. Liu W. Tian Y. Yang S. Zhou D. (2022). Identification and characterization of a novel homozygous splice site variant of PATL2 causing female infertility due to oocyte germinal vesicle arrest. Front. Genet. 13, 967288. 10.3389/fgene.2022.967288 36072676 PMC9441802

[B25] Varadi M. Anyango S. Deshpande M. Nair S. Natassia C. Yordanova G. (2022). AlphaFold protein structure database: Massively expanding the structural coverage of protein-sequence space with high-accuracy models. Nucleic Acids Res. 50 (D1), D439–D444. 10.1093/nar/gkab1061 34791371 PMC8728224

[B26] Wang W. Dong J. Chen B. Du J. Kuang Y. Sun X. (2020). Homozygous mutations in REC114 cause female infertility characterised by multiple pronuclei formation and early embryonic arrest. J. Med. Genet. 57 (3), 187–194. 10.1136/jmedgenet-2019-106379 31704776

[B27] Wang W. Wang W. Xu Y. Shi J. Fu J. Chen B. (2021). FBXO43 variants in patients with female infertility characterized by early embryonic arrest. Hum. Reprod. 36 (8), 2392–2402. 10.1093/humrep/deab131 34052850

[B28] Wu L. Chen H. Li D. Song D. Chen B. Yan Z. (2019). Novel mutations in PATL2: Expanding the mutational spectrum and corresponding phenotypic variability associated with female infertility. J. Hum. Genet. 64 (5), 379–385. 10.1038/s10038-019-0568-6 30765866

[B29] Xue N. Wang Y. Xu X. Jiang X. Li C. Wang J. (2022). Establishment of pluripotent stem cell line induced by PATL2 heterozygous mutation in patients with oocyte maturation defect-4. Stem. Cell. Res. 61, 102776. 10.1016/j.scr.2022.102776 35397397

[B30] Yang P. Yin C. Li M. Ma S. Cao Y. Zhang C. (2021). Mutation analysis of tubulin beta 8 class VIII in infertile females with oocyte or embryonic defects. Clin. Genet. 99 (1), 208–214. 10.1111/cge.13855 33009822

[B31] Zhang Z. Li B. Fu J. Li R. Diao F. Li C. (2020). Bi-Allelic missense pathogenic variants in TRIP13 cause female infertility characterized by oocyte maturation arrest. Am. J. Hum. Genet. 107 (1), 15–23. 10.1016/j.ajhg.2020.05.001 32473092 PMC7332649

[B32] Zheng W. Zhou Z. Sha Q. Niu X. Sun X. Shi J. (2020). Homozygous mutations in BTG4 cause zygotic cleavage failure and female infertility. Am. J. Hum. Genet. 107 (1), 24–33. 10.1016/j.ajhg.2020.05.010 32502391 PMC7332666

